# Protective Effects of Quercetin on Clothianidin-Induced Liver Damage in the Rat Model

**DOI:** 10.1155/2022/9399695

**Published:** 2022-01-18

**Authors:** Amin Gheshlaghi-Ghadim, Vahid Mohammadi, Elham Zadeh-Hashem

**Affiliations:** ^1^Department of Internal Medicine and Clinical Pathology, Faculty of Veterinary Medicine, Urmia University, Urmia, Iran; ^2^Department of Basic Sciences, Faculty of Veterinary Medicine, Urmia University, Urmia, Iran

## Abstract

Clothianidin (CTD) is a member of the neonicotinoid group of insecticides. This study was performed to determine the effect of quercetin on clothianidin-induced liver injury (CTD) in rats. Rats were randomly assigned to a normal control (saline), a CTD control-treated group (20 mg/kg) every 3 days for 21 days, and CTD + quercetin-treated groups (2.5, 5, and 10 mg/kg) for 35 days intraperitoneally. Enzyme activity, including alanine aminotransferase (ALT) and aspartate aminotransferase (AST), was measured by spectrophotometry in serum samples by an automatic biochemical analyzer using commercial kits. Total antioxidant capacity (TAC), malondialdehyde (MDA), and nitrate-nitrite were measured in homogeneous liver tissue samples of animals. A significant increase in ALT and AST enzyme activity was observed in the CTD group in comparison with that of the control groups. In the clothianidin + quercetin (10 mg/kg) group, the ALT and AST enzyme levels decreased compared to the clothianidin control group significantly (*P* < 0.05). The MDA value of the liver increased in the clothianidin-treated group compared to that of the control groups (*P* < 0.05). Decreased tissue TAC level was observed in the CTD-treated group in comparison with that of the control groups (*P* < 0.05). The MDA level of the liver decreased in the clothianidin + quercetin (10 mg/kg) group compared to that of the CTD control group (*P* < 0.05). Quercetin significantly raised the level of TAC in the liver tissue of the clothianidin + quercetin (10 mg/kg) treated group compared to that of the clothianidin control group (*P* < 0.05). Liver nitrate-nitrite measurement showed a significant increase in the clothianidin group compared to that of the normal control group (*P* < 0.05). Nitrate-nitrite level in the liver was decreased in clothianidin + quercetin (10 mg/kg) compared to that of the clothianidin control group significantly (*P* < 0.05). Histopathological investigation revealed that contact to the CTD induced tissue disorganization and inflammatory cell infiltration, but minor histopathological alterations in the liver tissues of rats treated with CTD and quercetin (10 mg/kg) were detected.

## 1. Introduction

A new category of toxins are neonicotinoids, which are potent insecticides that can be used for crop safety and ectoparasite control in companion animals [1]. Because of their low toxicity to animals, they have been gradually used worldwide, sometimes replacing organophosphate and carbamate pesticides [2]. The insecticidal action of neonicotinoids is attributed to their activity on nicotinic acetylcholine receptors (nAChRs) [3, 4]. The mechanism of neonicotinoids' acute toxicity is related to their effects on insects and mammalian nAChR by the nicotine agonist effect [5, 6]. According to the latest research, neonicotinoids show toxic effects on the reproductive, neurologic, hepatic, immune, and genetic systems of nontarget species [7–9].

Clothianidin (CTD; (E)-1-(2-chloro-1,3-thiazol-5-ylmethyl)-3-methyl-2-nitroguanidine) is a new widespread insecticide and a very active systemic and contact insecticide with low toxicity to mammals. CTD is one of the newest neonicotinoids, a class of synthetic organic pesticides. Due to its physical and chemical properties and high systemic root activity, CTD can be used in various useful strategies, including foliar, seed treatment, seed soaking, and soil application [10]. The CTD is an agonist of the nAChR, and it affects the regular neural signaling pathway by binding to the acetylcholine receptor on the postsynaptic membrane [4]. The neonicotinoids' beneficial toxicological characteristics are largely due to their target site selectivity [4]. Evidence revealed that CTD increases the levels of reactive oxygen species (ROS) and induces oxidative stress through the increase of glutathione peroxidase 4 (GPx4) [11].

Previous studies have demonstrated that some vegetables, fruits, and cereals contain the flavonoid quercetin (3,5,7,3,4-pentahydroxyflavanone). Quercetin can prevent lipid peroxidation by metal chelators and oxygen-free radical scavenger properties at in vitro and in vivo trials [12, 13]. It also has anticancer, antiulcer, anti-inflammatory, antiallergic, antiviral, and antibacterial effects, as well as heart protection and cataract prevention [14]. The antioxidant activity of quercetin has been linked to its positive effects in previous studies [15, 16]. To the author's knowledge, the role of quercetin during CTD toxicity has not been investigated. Then, the current study was designed to evaluate the potential protective effect of quercetin on liver function during chronic intraperitoneal injection of CTD on a rat model.

## 2. Materials and Methods

### 2.1. Compounds and Kits

CTD ((E)-1-(2-chloro-5-thiazolylmethyl)-3-methyl-2-nitroguanidine) and quercetin were purchased from Sigma-Aldrich (Germany). Kits for evaluation of total antioxidant capacity (TAC), malondialdehyde (MDA), and nitric oxide (NO) were purchased from Navand Salamat (Urmia, Iran) and Cib Biotech Co. (Tehran, Iran). The assay kits for Alanine aminotransaminase (ALT) and Aspartate aminotransaminase (AST) were purchased from Pars Azmoon (Tehran, Iran).

### 2.2. Animals and Trial Protocol

Male Wistar rats (*n* = 42, 180–200 g) were taken from the animal house care center of Urmia University. The temperature of the animal house was held at 24 ± 3°C with a 12 h light: 12 h dark set. The rats were fed a commercial pellet meal and water ad lib. The adaptation period was two weeks. Procedures were in agreement with the guidelines of the Ethical Board of Urmia University for animal studies (ethical code: UU-AEC-1216/pd/3).

The animals were randomly divided into six groups with seven animals in each group. The groups consisted of the control and the vehicle control groups which received sterile normal saline and dimethyl sulfoxide (DMSO), respectively. The third group received CTD at 20 mg/kg/day every three days for 21 days. Groups 4, 5, and 6 received CTD concurrent with 2.5, 5, and 10 mg/kg/day quercetin intraperitoneally every day consequently for 35 days, respectively [17]. After 24 h of the last dose administration, blood was withdrawn through a cardiac puncture under anesthesia. Ketamine (70 mg/kg body weight) and xylazine (5 mg/kg body weight) were used to anesthetize the animals intraperitoneally. Samples were centrifuged at 3,000 revolutions per minute for ten minutes, and sera were stored at −20°C till analysis. Following that, the liver was quickly separated and washed with normal saline and stored at −80°C.

### 2.3. Preparation of Tissue Homogenate

About 0.50 g of tissue pieces were transferred into the tube, and 2 mL of Tris-HCl buffer was added. They were then processed in 50 mM pH 7.0 phosphate-buffered saline (PBS) for 3 min at 14,000 rpm in a homogenizer (Ultra-Turrax, IKA Labortechnik, Germany). The homogenate was centrifuged for 30 min at 4°C. Samples were obtained from the supernatant for biochemical analysis.

### 2.4. Biochemical Assay

Serum activities of alanine aminotransaminase (ALT) and aspartate aminotransaminase (AST) were determined using automated biochemical analyzers (BT-1500, Biotecnica instruments, Italy) and diagnostic kits (Pars Azmoon, Tehran, Iran).

### 2.5. Total Antioxidant Capacity (TAC), Malondialdehyde (MDA) Levels, and Nitrate-Nitrite Assay

Total antioxidant capacity (TAC) and malondialdehyde (MDA) levels in the liver tissues were measured by a spectrophotometer (DANA-3200; Garni Medical Engineering Co., Tehran, Iran) using commercial assay kits (Navand Salamat, Urmia, Iran) following the manufacturer's instructions. Amounts of TAC and MDA in the liver homogenates were measured by the ferric reducing antioxidant power assay (FRAP) assay and the thiobarbituric acid reactive substance assay, respectively. The levels of nitrate-nitrite in the homogenates of the liver were measured colorimetrically using a commercial kit (Cib Biotech Co, Tehran, Iran) following the company's instructions [18].

## 3. Histopathological Examinations

Liver tissues were removed and fixed with 10% formalin embedded in paraffin for histopathological investigation by a light microscope. Stained hematoxylin and eosin slides were examined [13].

## 4. Statistical Analysis

Data analysis was done using one-way ANOVA followed by the Tukey test in SPSS software (SPSS Inc., Chicago, IL, USA). *P* < 0.05 was considered a significance level. Data were reported as the mean ± standard deviation.

## 5. Results

**Figure 1:**
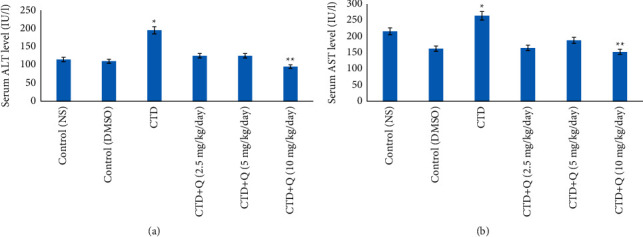
Effect of quercetin treatment against the toxic effects of CTD on serum hepatic biomarkers (a) alanine aminotransferase (ALT) and (b) aspartate aminotransferase (AST). ^*∗*^ indicates the significance from the control group at *P* < 0.05. ^*∗∗*^ indicates the significance from the CTD-treated group at *P* < 0.05. NS: normal saline; DMSO: dimethyl sulfoxide; CTD: clothianidin; Q: quercetin.

The levels of AST and ALT (unit/L) in comparison to those of control groups showed that clothianidin administration significantly raised serum levels of ALT and AST in the CTD group (*P* < 0.05). Moreover, simultaneous injection of quercetin ameliorated ALT and AST levels compared to that of the CTD (alone) treated group (*P* < 0.05; Figure 1).

### 5.1. Amounts of TAC and MDA

**Figure 2:**
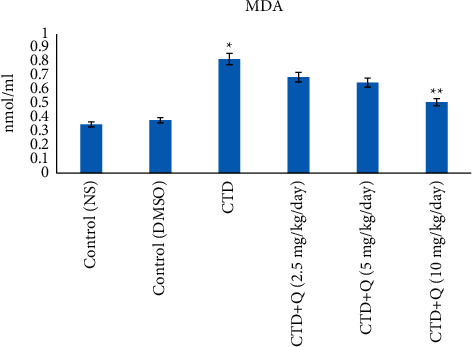
Comparison of malondialdehyde concentrations in the liver tissue of experimental groups. ^*∗*^ indicates the significance from the control group at *P* < 0.05. ^*∗∗*^ indicates the significance from the CTD-treated group at *P* < 0.05. NS: normal saline; DMSO: dimethyl sulfoxide; CTD: clothianidin; Q: quercetin; MDA: malondialdehyde.

**Figure 3:**
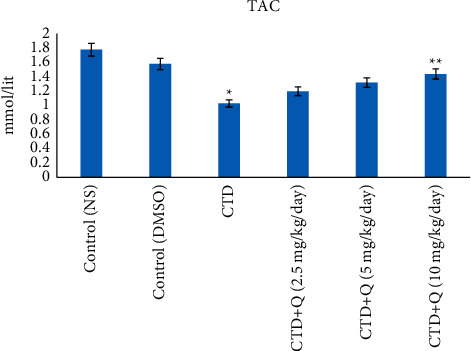
Total antioxidant capacity levels in the liver tissue of experimental groups. ^*∗*^ indicates the significance from the control group at *P* < 0.05. ^*∗∗*^ indicates the significance from the CTD-treated group at *P* < 0.05. NS: normal saline; DMSO: dimethyl sulfoxide; CTD: clothianidin; Q: quercetin; TAC: total antioxidant capacity.

The highest and lowest levels of MDA and TAC were detected in the CTD treated group, which differed compared to those of the control group (*P* < 0.05). Quercetin at 10 mg/kg decreased MDA levels and increased TAC amounts compared to those of the CTD treated group (*P* < 0.05). Amounts of TAC and MDA in the liver of quercetin-treatment groups (2.5 and 5 mg/kg) were not altered significantly compared to those of the CTD group (*P* > 0.05; Figures 2 and 3).

### 5.2. Amounts of Nitrate-Nitrite

**Figure 4:**
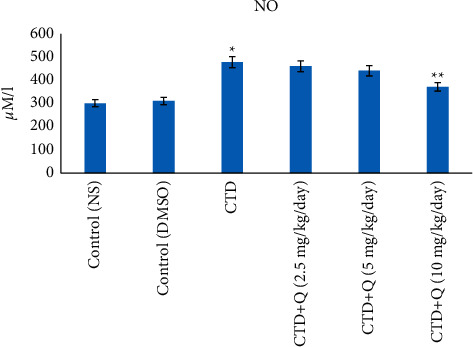
Nitrate-nitrate levels in the liver tissue of experimental groups. ^*∗*^ indicates the significance from the control group at *P* < 0.05. ^*∗∗*^ indicates the significance from the CTD-treated group at *P* < 0.05. NS: normal saline; DMSO: dimethyl sulfoxide; CTD: clothianidin; Q: quercetin.

The liver nitrate-nitrite amount revealed an important increase in the clothianidin group in comparison with the normal control group (*P* < 0.05). Moreover, at 10 mg/kg doses, the mean nitrate-nitrite level in the liver was considerably lower in the clothianidin + quercetin group compared to that of the clothianidin group (*P* < 0.05; Figure 4).

### 5.3. Histopathological Alterations in the Liver

**Figure 5:**
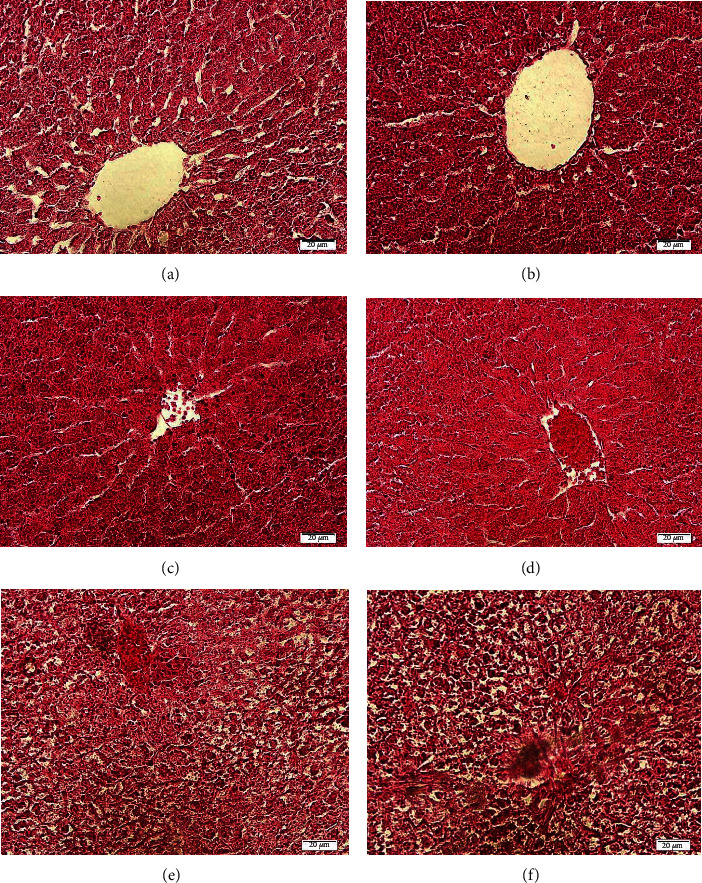
Representarive histologic micrographs of the liver samples in (a) control group, (b) vehicle group, (c) CTD group, (d) CTD + quercetin (2.5 mg/kg) group, (e) CTD + quercetin (5 mg/kg), and (f) CTD + quercetin (10 mg/kg) (H&E).

The histology of the liver in the control group was normal, and the liver tissue showed no abnormal alterations. When compared to the control group, the clothianidin-treated animals' livers revealed tissue disorganization, infiltration of inflammatory cells, bleeding, vacuolar degeneration, sinusoid dilatation, and necrosis. In the CTD + quercetin (10 mg/kg) group, the number of apoptotic and necrotic cells was reduced compared to the CTD group (Figure 5).

## 6. Discussion

In the present study, the protecting influence of quercetin was carried out against CTD-induced toxicity in the rat liver. Extensive use of neonicotinoids in agriculture can increase the toxic properties and side effects of insecticides and be lethal to humans as well as animals. Even low levels of pesticide residues on fruits and vegetables put consumers, especially children, at risk of cumulative exposure. In comparison to other insecticide classes, neonicotinoids have distinct physical and toxicological features. Because the symptoms of neonicotinoid poisoning are similar to those of nicotine poisoning, mediation of neonicotinoid toxicity was considered centrally in mammals [4].

Aminotransferases are enzymes that indicate the health and function of the liver. Biomarkers of liver injury include ALT and AST [19]. In this study, CTD caused a considerable increase in serum ALT and AST activity in rats, as well as a significant decrease in the CTD + quercetin (10 mg/kg) group. The increases in these enzymes in the serum could be attributed to tissue injury and eventual transfer of enzymes into the blood from the injured liver tissues, as well as an increase in cell membrane permeability [20]. Whenever the plasma membrane of a hepatocyte is disrupted, enzymes usually found in the cytosol are released into the blood. Their assessment in serum is a valuable tool for determining the severity and type of hepatocellular damage [21]. However, similarly, exposure to other types of pesticides, namely, chloroquine and imidacloprid, was also associated with a concomitant increase in the enzymatic levels of ALT and AST [22, 23].

The flavonoid quercetin has been proven to have excellent antioxidant and anti-inflammatory capacities in vivo. It has also been documented to possess antioxidant, antifungal, anticarcinogenic, hepatoprotective, and cytotoxic activity [24].

The generation of reactive oxygen (oxidative stress) and nitrogen (nitrosative stress) species appears to play a significant role in inducing the damage caused by the use of neonicotinoids on lipids, proteins, and DNA in vertebrates and invertebrates. In this regard, the role of oxidative stress and production of reactive species of oxygen and nitrogen on nerve, immune, liver, kidney, and reproductive damage has been investigated [1, 25, 26]. Neonicotinoids induce oxidative stress and produce free oxygen and nitrogen species, thereby causing toxic effects. CTD-induced oxidative stress has previously been observed in the liver of rainbow trout as elevated MDA levels [27]. In the present study, we observed a significant increase in the concentration of MDA in the liver tissue of the CTD (alone) treated group and a decrease in the clothianidin + quercetin (10 mg/kg) group, respectively. MDA is a well-known indicator of lipid peroxidation. Excessive production of reactive oxygen species (ROS), such as superoxide radicals (O_2_^−^), hydroxyl radicals, hydrogen peroxide (H_2_O_2_), and singlet oxygen, can harm cells by damaging polyunsaturated fatty acids in membrane lipids and also proteins or genetic elements. Amounts of MDA, as well as antioxidant capacity, have been key biochemical components in the detection of tissue damage following liver tissue poisoning.

The antioxidant capacity of liver tissue was shown to be reduced in the CTD group but elevated in rats given quercetin (10 mg/kg) in the current study. Consistent with these findings, some studies have shown antioxidant properties for quercetin. As a result, it seems that by reducing the production of ROS, quercetin has the antioxidant capacity, which can reduce MDA and increase TAC in the treatment group.

The results of this study indicated that, in comparison to the control group, the serum nitrate-nitrite level of the CTD group increased significantly. Moreover, serum nitrate-nitrite level in the CTD + quercetin (10 mg/kg) group compared to that of the control clothianidin group decreased significantly. It appears that intracellular oxidative stress increases nitrite oxide synthase, leading to increased nitrite production and decreased cell viability [28]. Owing to high oxygen consumption, mitochondrial dysfunction can increase the generation of free radicals in most body tissues, as well as nitrite oxide radicals, and owing to oxidative and nitrosative stress, it can cause tissue damage, particularly in the liver [29]. Quercetin treatment inhibits nitrate-nitrite increase and supports the hepatoprotective effect of quercetin in CTD-treated rats. It was reported that the administration of imidacloprid and thiacloprid via oxidative stress induction significantly increased the value of nitrotyrosine and nitrate-nitrite biomarkers in the brain and liver of rats. In addition, thiacloprid increases nitrate-nitrite levels in polymorphonuclear leukocytes and the plasma of thiacloprid-exposed rats [30].

On histopathological investigation, CTD-treated liver slices of rats showed changes in the appearance of hepatocytes and obvious vacuolar degeneration of the cytoplasm. The vacuolation may be owing to the holding of fluid inside the hepatocytes after what is termed hydropic degeneration or cloudy swelling which may be due to decreased energy needed to regulate membrane fluid transport, mild hypoxia, or a short duration of metabolic stress. The results showed that the number of hepatocytes in the clothianidin control group was significantly reduced and the size of the central vein was significantly increased compared with that of the normal control group [31].

Treatment with quercetin (10 mg/kg) resulted in moderate improvement in the histopathological injury of hepatic CTDs, while lower doses of quercetin were less effective in this regard. It seems that free radicals attack liver cells and cause parenchymal cell necrosis. These cells can induce inflammation in the liver, causing mononuclear inflammatory cells to damage tissues. Necrotic cells release proinflammatory mediators, which can worsen liver damage caused by poisons. Furthermore, the generation of free radicals and following oxidative stress may be one of the most serious and fundamental reasons for liver cell death [30]. However, quercetin (10 mg/kg) was almost able to prevent this from happening.

An extensive variety of biological activities such as anti-cancer, antiviral, anti-inflammatory, antibacterial, and antioxidant activities have been stated from quercetin [31]. Quercetin has been shown to scavenge free radicals and provide an antioxidant barrier by chelating divalent cations and preventing ROS formation [32, 33]. It has been known that quercetin not only distributes well in the water phase to scavenge free radicals but can also be anchored to the polar head of phospholipids and readily dispersed in the lipid bilayer of the cell membrane, protecting it from oxidative toxicity at the cellular level [34].

In conclusion, current research showed that quercetin has antioxidant properties. This is likely due to its enhancing effect on cellular antioxidant defenses, which may further contribute to its protective action against lipid peroxidation and protection against oxidative damage in CTD-induced hepatotoxicity.

## 7. Conclusion

Quercetin can partially prevent liver damage from CTD toxicity. The histopathological alterations of the liver further support this conclusion. In terms of public health, if our diet is likely to be contaminated by pesticides, we should eat more foods rich in quercetin [35].

## Data Availability

The data used to support the findings of this study are available from the corresponding author upon reasonable request.
